# Health-related quality of life of adolescents with type 1 diabetes mellitus

**DOI:** 10.1590/1518-8345.2961.3210

**Published:** 2019-12-05

**Authors:** Maria Amélia de Souza, Roberto Wagner Junior Freire de Freitas, Luciane Soares de Lima, Manoel Antônio dos Santos, Maria Lúcia Zanetti, Marta Maria Coelho Damasceno

**Affiliations:** 1 Universidade Federal de Pernambuco, Centro Acadêmico de Vitória, Vitória de Santo Antão, PE, Brazil.; 2 Fundação Oswaldo Cruz, Ceará, CE, Brazil.; 3 Universidade Federal de Pernambuco, Departamento de Enfermagem, Recife, PE, Brazil.; 4 Universidade de São Paulo, Faculdade de Filosofia, Ciências e Letras de Ribeirão Preto, Ribeirão Preto, SP, Brazil.; 5 Universidade de São Paulo, Escola de Enfermagem de Ribeirão Preto, PAHO/WHO Collaborating Centre for Nursing Research Development, Ribeirão Preto, SP, Brazil.; 6 Universidade Federal do Ceará, Fortaleza, CE, Brazil.

**Keywords:** Quality of Life, Diabetes Mellitus, Type 1, Adolescent, Adolescent Health, Nursing, Chronic Disease, Qualidade de Vida, Diabetes Mellitus Tipo 1, Adolescente, Saúde do Adolescente, Enfermagem, Doença Crônica, Calidad de Vida, Diabetes Mellitus Tipo 1, Adolescente, Salud del Adolescente, Enfermería, Enfermedad Crónica

## Abstract

**Objective:**

To evaluate the health-related quality of life of adolescents with type 1 diabetes mellitus, associating it with socio-demographic, clinical and biochemical variables.

**Method:**

Cross-sectional study with 92 adolescents with type 1 diabetes mellitus. A form containing socio-demographic, clinical and biochemical variables was used, as well as the Diabetes Quality of Life for Youths questionnaire. Descriptive statistics and logistic regression were used for analysis.

**Results:**

Regarding socio-demographic variables, economic class showed statistically significant differences in relation to total Health Related Quality of Life (p-value =0.02) and the impact domain (p-value =0.009). However, the impact domain was more compromised. Diabetes-related complications (p-value =0.004), number of hospitalizations (p-value =0.01), number of daily insulin injections (p-value =0.02), glycated hemoglobin (p-value =0.002) and triglycerides (p-value =0.03) were associated with greater impairment of quality of life related to total health and greater dissatisfaction.

**Conclusion:**

Single male adolescents with lower level of education and high glycated hemoglobin levels were more likely to have lower health-related quality of life.

## Abstract (pt)

**Objetivo:**

avaliar a qualidade de vida relacionada à saúde de adolescentes com diabetes *mellitus* tipo 1, associando-a às variáveis sociodemográficas, clínicas e bioquímicas.

**Método:**

estudo transversal, com 92 adolescentes com diabetes *mellitus* tipo 1. Utilizou-se formulário contendo variáveis sociodemográficas, clínicas e bioquímicas, e o Instrumento Qualidade de Vida para Jovens Diabéticos. Para análise utilizou-se estatística descritiva e regressão logística.

**Resultados:**

em relação às variáveis sociodemográficas, a classe econômica mostrou diferenças estatisticamente significantes entre os escores da Qualidade de Vida Relacionada à Saúde total (p-valor =0,02) e o domínio impacto (p-valor =0,009). No entanto, o domínio impacto se mostrou mais comprometido. As complicações relacionadas à doença (p-valor =0,004), número de internações (p-valor =0,01), número de aplicações diárias de insulina (p-valor =0,02), hemoglobina glicada (p-valor =0,002) e de triglicerídeos (p-valor =0,03) mostraram-se relacionadas ao maior prejuízo da qualidade de vida relacionada à saúde total e maior insatisfação.

**Conclusão:**

adolescentes do sexo masculino, solteiros, com menor nível de escolaridade e com níveis elevados de hemoglobina glicada apresentaram maiores chances de terem qualidade de vida relacionada à saúde rebaixada.

## Abstract (es)

**Objetivo:**

evaluar la calidad de vida relacionada con la salud de los adolescentes con diabetes *mellitus* tipo 1, asociándola con variables sociodemográficas, clínicas y bioquímicas.

**Método:**

estudio transversal con 92 adolescentes con diabetes mellitus tipo 1. Se utilizó un formulario que contenía variables sociodemográficas, clínicas y bioquímicas, así como el Instrumento Calidad de Vida para Jóvenes Diabéticos. Para el análisis, se utilizaron estadística descriptiva y regresión logística.

**Resultados:**

con respecto a las variables sociodemográficas, la clase económica mostró diferencias estadísticamente significativas entre los puntajes de la Calidad de vida Relacionada con la Salud total (p-valor =0,02) y el dominio de impacto (p-valor =0,009). Sin embargo, el dominio impacto resultó estar más comprometido. Las complicaciones relacionadas con la enfermedad (p-valor =0,004), número de hospitalizaciones (p-valor =0,01), número de aplicaciones diarias de insulina (p-valor =0,02), hemoglobina glucosilada (p-valor =0,002) y de triglicéridos (p-valor =0,03) se relacionaron con un mayor deterioro de la calidad de vida relacionado con la salud total y una mayor insatisfacción.

**Conclusión:**

los adolescentes varones solteros con un nivel educativo más bajo y niveles de hemoglobina glucosilada altos tenían más probabilidades de tener una calidad de vida más baja debido a la salud.

## Introduction

In recent years, there has been increasing interest in investigating chronic diseases in specific populations. One example is adolescents with type 1 diabetes mellitus (T1DM), considering that this condition involves complex treatment and long-term impairment of quality of life due to specific care needs^(^ 1 ^-^ 3 ^)^.

The care provided to adolescents with T1DM should consider the peculiarities of this age group, such as changes in insulin sensitivity related to sexual maturity and physical growth, ability to provide self-care, and neurological vulnerability to hypoglycemia and possibly hyperglycemia^(^ 4 ^)^. With the technological and therapeutic advances, as well as new knowledge about the psychological and social factors related to the disease, T1DM treatment is currently based on the triad insulin, monitoring and education on diabetes (which includes healthy eating and regular physical activity). In this regard, attention to family dynamics is also essential for a successful treatment plan^(^ 4 ^)^. As these demands continue throughout life, the complexity of the intensive care plan may compromise quality of life.

Adolescents with chronic diseases are exposed to potential stressors that affect their quality of life^(^ 2 ^,^ 5 ^)^. The presence of a chronic condition, such as T1DM, during this period of human development has a psychosocial impact, since it leads to changes in multiple areas of personal and social identity, increasing the risks of developing emotional and behavioral disorders^(^ 6 ^)^. These disorders can be triggered by Diabetes Distress, which refers to negative emotions arising from living with the disease and the burden of self-management of diabetes^(^ 7 ^)^.

The prevalence of distress is well documented among adults, but not among the adolescent population^(^ 7 ^)^. A systematic review showed that around one third of adolescents with T1DM experience elevated diabetes distress, frequently associated with suboptimal glycemic control, low self-efficacy and reduced self-care, which compromises their quality of life^(^ 7 ^)^. The intrinsic challenges of adolescence and the way this population handles the disease in their daily lives affect disease management and create needs that must be appreciated by health care professionals in order to provide self-care support and help improving quality of life^(^ 8 ^)^.

Due to the need for a concept that favors dialogue among health professionals, the World Health Organization (WHO) defined quality of life as “an individual’s perception of their position in life in the context of the culture and value systems in which they live and in relation to their goals, expectations, standards and concern”^(^ 9 ^)^. In particular, the concept of Health-Related Quality of Life (HRQoL) is related to an individual’s perceived physical and mental health. Moreover, it involves the perception of the impact of a disease and its treatment, according to the ability of the person to develop their potential and lead a full life^(^ 10 ^)^.

Despite the significant number of scientific studies on T1DM in children and adolescents in international^(^ 11 ^-^ 12 ^)^ and national literature^(^ 8 ^,^ 13 ^-^ 14 ^)^, there are only few studies investigating its relationship with socio-economic, clinical and biochemical characteristics. These aspects may affect the treatment and self-management of the disease and, consequently, compromise the quality of life of adolescents with T1DM.

Given the above, the following research question was elaborated: “Are the HRQoL domains related to the socio-demographic, clinical and laboratory characteristics of adolescents with T1DM?” Based on the assumption that these variables may affect the HRQoL of adolescents with T1DM, this study aimed to evaluate the HRQoL of adolescents with T1DM, associating it with socio-demographic, clinical and laboratory variables.

The results obtained will hopefully increase knowledge and help promoting quality of life, considering the particularities of adolescents and their individual characteristics and social context, as well as the glycemic control goals for this phase of life, in accordance with current diabetes education guidelines.

## Method

Cross-sectional study conducted at a DM treatment service located in Recife-PE, Brazil, from January to July 2014.

The universe of the study was composed of 120 adolescents with T1DM who regularly attended the service, of which 28 were no longer in regular care, due to having more than three consecutive absences. Thus, the population consisted of 92 adolescents with T1DM, of both genders, between 10 and 19 years old and in regular follow-up at the referred service. The definition of adolescence used here was based on the period of human development established by the World Health Organization (1975), which is adopted in most studies. The Diabetes Quality of Life for Youths (DQOLY) was used.

Participants were recruited while in the waiting room of the hospital. The adolescents and their parents or guardians were invited to participate in the research while awaiting consultation with the pediatric endocrinologist. The nature and the objectives of the study were explained. Participants aged 18 years and over signed the Informed Consent Term (ICT). Minors signed the Informed Agreement Term (IAT) and their parents or legal guardians signed the ICT.

The socio-demographic variables assessed were: gender, age, skin color/ethnicity, civil status, education and economic class. Economic class was determined based on the Brazil’s Economic Classification Criteria, elaborated by the National Association of Research Companies. Clinical variables included: time since diagnosis, onset of first symptoms, diseases associated with T1DM and chronic complications, hospitalizations in the last year, drug treatment, number of daily insulin injections, self-monitoring of blood glucose at home, frequency of self-monitoring, chronic complications of diabetes, hypoglycemia in the last month, frequency of hypoglycemia in the last month, hyperglycemia in the last month and, finally, frequency of hyperglycemia in the last month.

Biochemical variables included: preprandial glucose, postprandial glucose, glycated hemoglobin (HbA1c), high-density lipoprotein - HDL, low-density lipoprotein - LDL, triglycerides and total cholesterol level. The Diabetes Quality of Life for Youths (DQOLY) was used to evaluate the variable quality of life.

A form elaborated by the researchers and completed through consultation to medical records was used to collect socio-demographic, clinical and biochemical variables. The most recent value registered in the medical records was considered for the collection of laboratory test results. The DQOLY, translated, validated and culturally adapted to Brazil, based on the The Diabetes Quality of Life for Youths (DQOLY), was used to measure the variable quality of life^(^ 15 ^)^.

The instrument validation process showed adequate psychometric properties. Reliability was assessed by internal consistency and test-retest. Cronbach’s alpha coefficients of the validated instrument were: 0.8695 for the satisfaction domain; 0.8658 for the impact domain; 0.8387 for the worries domain and 0.93 for the total score^(^ 15 ^)^.

Data were collected by one of the researchers of the team responsible for the study, who was previously trained for this activity. The adolescents were asked to answer the DQOLY questions individually, to avoid interference from parents or guardians in the answers. No participants declined the invitation to complete the instrument. The time of application was approximately 20 minutes

The DQOLY is composed of 51 items distributed in three dimensions satisfaction, impact and worries, with 17, 23 and 11 items, respectively. The questions use a 5-point Likert scale ranging from very satisfied (score 1) to very dissatisfied (score 5) in the satisfaction domain and from never (score 1) to always (score 5) in the impact and worries domains. The total scores and scores by domain were calculated based on the sum of the items. Lower scores were associated with better HRQoL and higher scores with worst HRQoL score, except for item B7 of the impact domain, which is inverted. In addition, a question addressing adolescents’ perceptions of their health status compared to healthy peers was included and had only four response options: excellent, good, satisfactory, and poor(15).

Statistical analysis of the data was performed using the International Business Machines Corporation (IMB) Statistical Package for the Social Sciences (SPSS) Statistics for Windows, version 20.0. Absolute and relative numbers, measures of central tendency and variability were used to present the exploratory analysis. The Student’s t-test, Analysis of Variance (ANOVA) and Pearson’s correlation coefficient were used for statistical inference. A confidence interval of 95% and a significance level of 5% were adopted in order to make proper inferences based on the interpretation of the information. The reliability of the instrument was analyzed by Cronbach’s α coefficient.

Univariate analysis of possible associations between socio-demographic variables and HRQoL and its domains was performed using Student’s t-test and ANOVA for the economic class variable, and Tukey’s post hoc test for the total HRQoL. For clinical variables, inferential analysis was used to compare the number of daily insulin injections with total HRQoL scores and scores by domain.

In the bivariate analysis, the quality of life score in each domain was categorized as low/very low or high/very high. Low HRQoL was considered to determine the adjusted values ​​of the variables in the logistic regression model, the odds ratios and their respective confidence intervals at 95%. The variables used in the adjustment showed significant odds ratios for quantifying risk of presenting low HRQoL.

The cut-off scores to classify the HRQoL level as very high, high, low and very low were defined in quartiles. For example, the domain satisfaction ranges from 17 to 85. Thus, there are 85 - 17 = 69 units between the minimum and the maximum value. Divided by four (quartiles), we will have 69/4 = 17.25 (approximately 17). Thus, the 1st quartile includes the values ​​between the minimum value (17), and the minimum value plus the size of the interval (69/4 = 17,25 = 17). Thus, the first quartile is between 17 and 34 (17 + 17 = 34). The second quartile includes the values between 35 and 34 + 17 = 51, and so on, following this logic for the other intervals (Table 1).

**Table 1:** Distribution of Total Health-Related Quality of Life levels and domains of adolescents with T1DM*. Recife, PE, Brazil, 2014

Quality of Life levels	Domains			
	Satisfaction	Impact	Concerns	Total
Very high	17 - 34	22 - 44	11 - 22	50 - 100
High	35 - 51	45 - 66	23 - 33	101 - 150
Low	52 - 68	67 - 88	34 - 44	151 - 200
Very low	69 - 85	89 - 110	45 - 55	201 - 250

* T1DM = Type 1 Diabetes Mellitus

A Logistic Regression Model with three explanatory variables (socio-demographic, clinical and biochemical) and one outcome (the HRQoL of adolescents with T1DM) was applied for the multiple analysis. When more than one variable is adjusted at the same time in a logistic regression model, the estimation process of the model seeks to select the smallest possible set of explanatory variables, i.e., which variables best explains the outcome variable, which in the case is low HRQoL.

The risk of adolescents having low or high HRQoL total score or low or high score by domain was calculated by the odds ratio (OR), as a function of explanatory variables (socio-demographic, clinical and biochemical variables) and of the outcome variable (DQOLY domain scores and total score). The outcome variable was divided into only two categories: 0 - “low”, equivalent to the original categories “low” and “very low”; and 1 - “high”, equivalent to the original categories “high” and “very high”. The “low” category of the outcome variable is associated with low DQOLY scores, and therefore is associated with high HRQoL. The “high” category - associated with the outcome variable - is associated with the instrument’s high scores, which indicates low HRQoL. The project was approved by the Research Ethics Committee, process No. 165.227 / 2012.

## Results

Of the 92 (100%) adolescents, 50 (54.3%) were male. The mean age was 14.6 years, with standard deviation (SD) of 2.9. As for skin color/ethnicity, 46.7% were white. Most participants were single (96.7%). Regarding the level of education, 53.3% were in elementary school. Equivalent proportions of participants belonged to economic classes B2, C1 and C2.

The mean time since diagnosis was 6.8 years (SD=4.5 years) and the mean age at onset of the first symptoms was 7.6 years (SD=4 years). It was found that 96.7% had no record of diseases associated with T1DM and 87% had no chronic complications. One third of the adolescents had not been hospitalized at least once in the previous year. Regarding drug treatment, most adolescents (59.8%) used four or more insulin injections per day. Among the participants, 88% performed self-monitoring of blood glucose every day, and 51.1% performed it between one and three times. The mean frequency of hypoglycemia and hyperglycemia exceeded two episodes in the month preceding data collection. Only 18.5% of the adolescents had normal glycated hemoglobin (HbA1c) levels, 25% had normal preprandial glucose level and 18.5% had normal postprandial glucose level.

The reliability of the instrument in the studied population, calculated through Cronbach’s α coefficient, was 0.85. The results of the total HRQoL scores and scores by domain after the application of the DQOLY are presented in Table 2. The total HRQoL mean scores and the scores by domains (satisfaction, impact and worries) are close to the minimum scores, which represents high HRQoL. The impact domain had the highest mean value (53.0), indicating a low HRQoL.

**Table 2:** Distribution of total quality of life scores and scores by domain of adolescents with T1DM*. Recife, PE, Brazil, 2014

Variables	Mean	Standard Deviation	Minimum	Maximum
Total quality of life	117.5	20.1	79.0	174.0
Satisfaction domain	38.6	9.3	20.0	65.0
Impact domain	53.0	10.4	34.0	84.0
Worries domain	25.8	6.6	13.0	40.0

* T1DM = Type 1 Diabetes Mellitus

The items with the highest scores in each domain were: satisfaction (A2, A5, A6), impact (B20, B21, B22) and worries (C3, C5, C7), as shown in Table 3.

**Table 3:** Distribution of the items with highest scores in the DQOLY* by domain. Recife, PE, Brazil, 2014

DQOLY Domains*	Items	Mean ± SD^†^ Median - Mode (1 - 5)
**Satisfaction**	*A2-* How satisfied are you with the time you spend doing lab and eye exams? *A5-* How satisfied are you with the flexibility of your diet? *A6-* How satisfied are you with the burden your diabetes places on your family?	2.7±1.1 3-2 2.9±1.2 3-2 2.6±1.1 3-2
**Impact**	*B20-* How often do you feel that your parents are too protective? *B21*- How often do you feel that your parents worry too much about your diabetes? *B22*- How often do you feel that your parents act as if they, not you, have diabetes?	4.4±0.9 5-5 4.6±0.8 5-5 3.5±1.5 4-5
**Concerns**	*C3*- How often do you worries about whether you will get the job you want? *C5*- How often do you worries about whether you will complete your education? *C7*- How often do you worries about whether you will get complications from your diabetes?	3.1 ±1.4 3-3 3.7±1.4 4-5 3.1±1.6 3-5

* DQOLY = The Diabetes Quality of Life for Youths;

† SD = Standard Deviation

Among the socio-demographic variables, economic class showed statistically significant differences in relation to total HRQoL scores (p-value=0.02) and the impact domain (p-value=0.009). In this domain, it was found that economic class B2 (M=48.1) differs from classes C2 and D, which have higher scores (M=57.2 and M=61.4, respectively), suggesting worse quality of life. When comparing total HRQoL between classes D and C2, scores were lower among participants in class D (M=132.2) when compared to those in class B2 (M=108.0).

Regarding clinical variables, specifically the presence of complications related to T1DM, there were statistically significant differences in relation to total HRQoL score (p-value=0.004) and the impact domain (p-value=0.002). These results indicate greater impairment of total HRQoL and greater impact on the daily lives of patients when they have complications associated with the disease.

The occurrence of hospitalizations in the previous year presented statistically significantly differences in relation to the total HRQoL score (p-value =0.01) and the domains satisfaction (p-value =0.01) and worries (p-value =0.02). Participants who had had one or more hospitalizations had higher scores, suggesting greater impairment of total HRQoL. In addition, participants who had been hospitalized in the previous year presented greater dissatisfaction and worries scores.

The number of insulin injections presented statistically significant differences in relation to the total HRQoL score (p-value =0.02) and the satisfaction domain (p-value =0.01). These results show that adolescents who used four or more insulin injections per day presented greater impairment of total HRQoL and greater dissatisfaction. Similar results were found for the self-monitoring variable, which presented statistically significant differences in relation to total HRQoL (p-value =0.03) and the satisfaction domain (p-value =0.02).

Regarding the biochemical variables, the bivariate analysis showed statistically significant differences in the scores of the worries domain in relation to the variables glycated hemoglobin and postprandial glucose, p-value =0.002 and p-value =0.02, respectively. This result suggests that adolescents who presented poor glycemic control had higher scores in the worries domain. Statistically significant differences were also found in total HRQoL scores in relation to triglyceride levels (p-value =0.03). Statistical analysis showed that participants with higher triglyceride values ​​presented greater impairment of total HRQoL.

Regarding self-perceived health compared to healthy peers, it was found that 43.5% of the participants considered it good and 19.6% poor. Statistically significant differences were identified in relation to the total HRQoL scores and the three domains. These results indicate that adolescents who rated their health as poor presented greater impairment of total HRQoL.

Table 4 shows the logistic regression model with adjusted values of the odds ratios for the socio-demographic, clinical and biochemical variables of adolescents, as a function of their HRQoL. It should be noted that a score equal to 1 in each response variable of the DQOLY and its three domains determined low HRQoL.

**Table 4:** Evaluation of socio-demographic variables associated with health-related quality of life of adolescents with T1DM*. Recife, PE, Brazil 2014

Variables	Parameters	Domains of the Diabetes Quality of Life for Youths questionnaire							
		Satisfaction		Impact		Worries		Total	
		OR^†^	95% CI^‡^	OR*†*	95% CI^‡^	OR^†^	95% CI^‡^	OR^†^	95% CI^‡^
Gender	Female (R)^§^	**--**	--	**--**	--	**--**		**--**	
	Male	**9.0**	3.63 22.7	**7.3**	3.1 17.2	**11.5**	4.1 31.9	**11.5**	4.1 31.5
Color/ethnicity	Yellow (R)^§^	**--**		**--**	--	**--**	--	**--**	
	White	**7.6**	3.0 19.3	**6.2**	2.6 14.6	**6.0**	2.5 14.2	**13.0**	4.0 42.1
	Black	**--**		**--**	--	**--**	--	**--**	
	Brown	**8.5**	3.0 23.9	**8.5**	3.0 23.9	**8.5**	3.0 23.9	**11.7**	3.6 37.9
Civil status	Married/cons. union (R)^§^	**--**	--	**--**	--	**--**	--	**--**	
	Single	**10.1**	4.9 20.9	**7.9**	4.1 15.2	**7.8**	4.0 15.1	**13.7**	6.0 31.3
Level of Education	Higher Education (R)^§^	**--**	--	**--**	--	**--**		**--**	
	Elementary Education	**7.3**	3.1 17.2	**7.3**	3.1 17.2	**7.3**	3.1 17.2	**9.0**	3.6 22.7
	Secondary Education	**12.7**	3.9 41.0	**9.2**	3.3 25.9	**9.0**	3.2 25.3	**39.0**	5.4 283.9

* T1DM = Type 1 Diabetes Mellitus;

† OR = Odds Ratio adjusted based on the logistic regression model. All OR values in bold are significant at 5%;

‡ C.I. = Confidence Interval;

§ R = comparative reference for odds ratio interpretation

For the satisfaction domain, it was found that men are nine times more likely (OR = 9,000) to have low HRQoL (i.e. high score in the response variable) than women. Adolescents who are single are 10 times more likely to have low HRQoL than those who are married or in a consensual union. Participants in elementary and secondary education are approximately 7.3 and 12.7, respectively, more likely to have low HRQoL compared to adolescents in higher education (Table 4).

The evaluation of biochemical factors revealed that adolescents with high HbA1c levels are 11 times more likely to have low total HRQoL. Adolescents with high pre and postprandial glucose levels were approximately eight times more likely to have low HRQoL. Finally, adolescents with abnormal triglycerides ​​were 5.5 times more likely to have low HRQoL. Similar results were found for the impact and worries domains, as well as for total HRQoL (Tables 4 and 5).

**Table 5:** Evaluation of clinical and biochemical variables associated with health-related quality of life of adolescents with T1DM*. Recife, PE, Brazil 2014

Variables	Parameters	Domains of the Diabetes Quality of Life for Youths questionnaire							
		Satisfaction		Impact		Worries		Total	
		OR^†^	95% CI^‡^	OR^†^	95% CI^‡^	OR^†^	95% CI^‡^	OR^†^	95% CI^‡^
Medication: intermediate	No (R)^§^	**--**	--	**--**	--	**--**	--	**--**	--
	Yes	**8.3**	3.8 18.1	**7.1**	3.4 14.9	**9.7**	4.2 22.4	**15.0**	5.4 41.3
Medication (dose): fast acting	No (R)^§^	**--**	--	**--**	--	**--**	--	**--**	--
	Yes	**6.6**	3.0 14.5	**5.6**	2.6 11.9	**16.3**	5.1 52.4	**12.0**	4.3 33.3
Medication (dose): slow acting	No (R)^§^	**--**	--	**--**	--	**--**	--	**--**	--
	Yes	**25.0**	3.4 184.5	**12.0**	2.8 50.8	**7.7**	2.3 25.5	**25.0**	3.4 184.5
Medication (dose): ultra fast acting	No (R)^§^	**--**	--	**--**	--	**--**	--	**--**	--
	Yes	**28.0**	3.8 205.8	**13.5**	3.2 56.8	**6.2**	2.2 18.0	**28.0**	3.8 205.8
Glycated hemoglobin	Normal (R)^§^	**--**	--	**--**	--	**--**	--	**--**	--
	Altered	**8.4**	4.0 17.4	**8.4**	4.0 17.4	**6.4**	3.3 12.5	**11.3**	5.0 26.1
Preprandial glucose	Normal (R)^§^	**--**	--	**--**	--	**--**	--	**--**	--
	Altered	**8.9**	4.0 19.3	**6.7**	3.3 13.4	**8.7**	4.0 19.0	**10.3**	4.5 23.9
Postprandial glucose	Normal (R)^§^	**--**	--	**--**	--	**--**	--	**--**	--
	Altered	**8.4**	4.0 17.4	**7.3**	3.6 14.7	**8.2**	4.0 17.2	**11.3**	5.0 26.1
Total Cholesterol	Desejável (R)^§^	**--**	--	**--**	--	**--**	--	**--**	--
	Borderline	**16.0**	2.1 120.6	**--**	--	**7.5**	1.7 32.8	**--**	--
	High	**6.4**	2.5 16.4	**6.4**	2.5 16.4	**11.3**	3.5 36.9	**11.3**	3.5 36.9
High-Density Lipoprotein	Optimal (R)^§^	**--**	--	**--**	--	**--**	--	**--**	--
	Above Optimal	**12.2**	4.9 30.4	**12.2**	4.9 30.4	**8.3**	3.8 18.1	**64.0**	8.9 461.3
Low-Density Lipoprotein	Optimal (R)^§^	**--**	--	**--**	--	**--**	--	**--**	--
	Borderline	**7.3**	2.2 24.5	**7.3**	2.2 24.5	**7,3**	2.2 24.5	**24..0**	3.2 177.4
	High	**10.0**	1.3 78.1	**--**	--	**--**	--	**--**	--
Triglycerides	Optimal (R)^§^	**--**	--	**--**	--	**--**	--	**--**	--
	Borderline	**5.5**	1.2 24.8	**12.0**	1.6 92.3	**--**	--	**12.0**	1.6 92.3

* T1DM = Type 1 Diabetes Mellitus;

† OR = Odds Ratio adjusted based on the logistic regression model. All OR values in bold are significant at 5%;

‡ C.I = Confidence Interval;

§ R = comparative reference for odds ratio interpretation

According to Tables 4 and 5, socio-demographic, clinical and biochemical variables are associated with total HRQoL scores and scores by domain for the adolescents with T1DM investigated.

## Discussion

This study showed that the mean HRQoL scores and its domains were close to the minimum scores, indicating that, in general, the investigated adolescents with T1DM have high HRQoL. However, single, male participants with elevated glycated hemoglobin levels are more likely to have low HRQoL. Therefore, when assessing the HRQoL of adolescents with T1DM in outpatient follow-up, it is necessary to consider the evidence presented here to support diabetes education programs focused on the characteristics peculiar to this stage of development and on the promotion of quality of life. These results confirm the originality of this study and its contribution to the advancement of knowledge necessary to support the educational process^(^ 4 ^)^.

The results regarding total HRQoL and its domains among adolescents living in the Northeast region was similar to the results found in a study conducted in the Southeast region^(^ 15 ^)^. This finding suggests that even in a country with continental dimensions and multicultural historical and social formation such as Brazil, HRQoL perception remains similar in different regions.

A study with Portuguese adolescents with DM found high HRQoL, in contrast to research in eastern countries, which found that HRQoL ranged from moderate to low^(^ 16 ^)^. Regarding the items with the highest scores in each domain, dissatisfaction was related to the time spent in laboratory and eye exams, the flexibility of the diet and the burden the disease places on family dynamics. In the impact domain, the highest items were related to parents’ overprotective attitude and their excessive concern with their children’s glycemic control. Therefore, educating family members on how to effectively solve problems and resolve conflicts can support diabetes management and optimize glycemic control, reducing diabetes distress and, consequently, improving quality of life^(^ 17 ^)^. This process must continue throughout childhood and adolescence.

In the concern domain, the item with the highest value was related to ability to complete their education. These results suggest excessive care of parents and insecurity of adolescents regarding their personal competence to complete their education and the possibility of facing situations of discrimination in the school environment; these results are backed up by other research on the subject^(^ 3 ^,^ 8 ^)^.

When analyzing the high HRQoL observed among adolescents with T1DM in this study, it is necessary to consider that the participants were in a reference center for diabetes care, receiving assistance from a multidisciplinary team composed of nurses, doctors, nutritionists, dentists, psychologists, social workers and physical educators. This center also provides support resources such as: therapeutic groups for adolescents with T1DM, an experimental kitchen to teach dietary cooking, and a cardiopulmonary rehabilitation project, with the promotion of physical activity.

The results of the self-perceived health of adolescents with DM compared to their healthy peers revealed that those who rated their HRQoL as poor also had greater impairment of total HRQoL. This fact indicates that, despite of the chronicity of diabetes and the nuances of T1DM management, the investigated adolescents perceived themselves as healthy. These results show the need for permanent adaptation in order to appropriately manage the disease. Thus, adolescents are confronted with the need to constantly adjust and assimilate aspects related to the health-disease-care process and the impact of this condition on their daily lives. Positive association between HRQoL and self-perceived health was also reported in another study^(^ 15 ^)^. The results show the importance of offering support to patients with T1DM, guiding them in appropriate treatment to reduce the risk of acute and chronic complications and improve the quality of life and encouraging constant engagement in education programs^(^ 4 ^)^.

Multiple analysis indicated that HRQoL in adolescents with T1DM is associated with gender. A review study showed that girls with the disease experience more coercive control associated with culture and family when compared to boys, which has been shown to be a negative factor in relation to adolescents’ quality of life^(^ 3 ^)^. Another study also pointed out that girls reported greater impact and worries than boys^(^ 11 ^)^. The study found that female adolescents have better HRQoL in all dimensions of the DQOLY scale, except for the impact domain^(^ 16 ^)^.

The relationship between HRQoL and economic class was investigated in a study conducted in Germany, which found that low socioeconomic status was significantly associated with suboptimal T1DM management, despite of health care being freely available^(^ 18 ^)^. This may negatively affect quality of life. The present study showed statistically significant differences in total HRQoL (p-value =0.02) and in the impact domain (p-value =0.009). However, another research found no significant association between socioeconomic status and quality of life of adolescents^(^ 11 ^)^. Children and adolescents with T1DM who belong to lower socio-economic groups may be more susceptible to complications resulting from the disease^(^ 18 ^)^. In addition, the higher frequency of diabetes complications contributes to reduced life expectancy and lower HRQoL, as shown in the bivariate analysis conducted in this study.

The association between disease duration, treatment time and HRQoL has been addressed in national and international studies. In this study, the bivariate and multivariate analyzes with these variables did not find statistically significant differences, which is corroborated by other authors^(^ 11 ^,^ 16 ^,^ 19 ^)^. However, a similar study found significant association^(^ 12 ^)^, suggesting that these variables should be investigated in future studies. Age at diagnosis was not significantly associated with total HRQoL and its domains, which is in agreement with another study^(^ 11 ^)^.

The results presented here represent an advance in knowledge about HRQoL in adolescents with T1DM and indicate that certain individual characteristics and glycemic control factors are associated with perceived quality of life. Adjustments in the logistic regression provided evidence on how to quantify the risk of low quality of life among these adolescents, considering that risk factors (gender, civil status, education and glycated hemoglobin) may influence HRQoL in adolescents with T1DM. This study also found statistically significant differences in all domains of the scale, as well as in the total HRQoL score. The protective effects of intensive T1DM treatment are recognized, despite of the increase in HbA1 values ​​over the years of follow-up. However, there is still a gap between clinical evidence and daily practice, as the therapeutic goal is difficult to achieve and maintain throughout disease progression This is due to the numerous barriers involved in diabetes management, such as the occurrence and fear of hypoglycemic episodes, the complexity of treatment and the daily routine and the need for frequent monitoring and adjustment of insulin doses, factors that affect the quality of life of adolescents^(^ 4 ^)^.

The results confirm the importance of evaluating HRQoL of adolescents with T1DM and indicate possible associated factors, supporting the planning of health prevention and promotion actions. In addition, this study may lead to changes in healthcare, changing the perspective of health professionals to include aspects beyond the biological dimension of care. Understanding the effects of T1DM on HRQoL is also relevant for health managers and for public policies aimed at improving the quality of life of adolescents with T1DM.

Despite of the statistically significant associations between HRQoL and number of hospitalization, presence of complications, number of daily insulin injections, home self-monitoring and biochemical results, it is believed that the first four variables are strictly related to low quality of life of adolescents with diabetes due to their specific characteristics and immediate consequences. Those who used four or more insulin injections per day showed greater impairment of HRQoL and greater dissatisfaction^(^ 20 ^)^, as well as those who did not perform self-monitoring. These results corroborate a previous study that suggests that measures originally designed for adults may not be sufficiently sensitive to the concerns encountered in the daily lives of adolescents with T1DM. Thus, interventions to reduce diabetes distress in the short term and promote quality of life should involve strategies such as cognitive restructuring, goal setting, and problem solving^(^ 7 ^)^.

When adolescents are hospitalized, they feel deprived of the company of friends and have to be away from the school environment and apart from the family, while experiencing feelings of pain, anguish and sadness, which may compromise their HRQoL. Similar results were found in a study in which a bivariate analysis showed that quality of life was significantly associated with the number of hospitalizations^(^ 20 ^)^. On the other hand, a Portuguese study found that adolescents with better quality of life had a higher number of hospital admissions^(^ 16 ^)^.

A multicenter study found that the lower the glycated hemoglobin levels, the better the HRQoL, pointing to a strong association between improved quality of life and glycemic control. This study also pointed out that potentially modifiable behavioral factors related to glycemic control may contribute to the implementation of clinical interventions to improve HRQoL^(^ 21 ^)^.

Other studies should explore the socio-demographic, clinical and laboratory variables related to HRQoL among adolescents with T1DM to advance the knowledge of this topic. Limitations of the present study include the inability to generalize results and compare data due to study design and the cultural and regional differences that may affect quality of life, including the characteristics of the health service investigated.

Some findings may inspire future investigations based on new hypotheses and variables, such as: the significant number of adolescents who presented hypoglycemic and hyperglycemic episodes, low adherence to blood glucose monitoring, hospitalization rates and disease management tests outside normal range, difficulty to follow the diet plan, concern for the professional future and parental overprotection.

## Conclusion

The results of this study allowed concluding that the mean HRQoL scores and the domains satisfaction, impact and worries were close to the minimum scores, indicating that adolescents with T1DM have high HRQoL. However, the impact domain was the most compromised. Regarding socio-demographic variables, adolescents with T1DM belonging to the economic class D presented greater impairment in HRQoL. Regarding clinical and biochemical variables, complications related to the disease, number of hospitalizations, number of daily insulin injections, glycemic control and triglycerides were associated with greater impairment of total HRQoL and greater dissatisfaction. Adolescents who evaluated their health as poor showed greater impairment of total HRQoL. In the logistic regression analysis, single male adolescents, with lower level of education and with high HbA1c levels were more likely to have low HRQoL.

This study provides evidence that can promote better quality of life considering the particularities of adolescents with T1DM, their individual characteristics and social context, as well as the glycemic control goals for this stage of development, according to current diabetes education guidelines.
